# The *ATRA-21* gene-expression model predicts retinoid sensitivity in *CEBPA* double mutant, *t(8;21)* and *inv(16)* AML patients

**DOI:** 10.1038/s41408-019-0241-5

**Published:** 2019-09-30

**Authors:** Marco Bolis, Mineko Terao, Linda Pattini, Enrico Garattini, Maddalena Fratelli

**Affiliations:** 1 Laboratory of Molecular Biology, IRCCS-Istituto di Ricerche Farmacologiche “Mario Negri”, Milano, Italy; 2 Department of Electronics, Information and Bioengineering, Politecnico di Milano, Milano, Italy

**Keywords:** Medical research, Cell biology

Dear Editor,

ATRA is the first example of clinically effective targeted antitumor agent and it is part of the standard protocol used in the treatment of acute promyelocytic leukemia (APL)^1^. The mechanisms underlying the therapeutic activity of ATRA and classic chemotherapeutic agents are different^2^. In fact, the retinoid is primarily a differentiating/antiproliferative agent and it is largely devoid of direct cytotoxic activity against the leukemic cell. This is at the basis of the low systemic toxicity associated with ATRA in the clinics and it justifies the interest in the use of the retinoid in oncologic contexts other than APL. As for the last aspect, in the field of onco-hematology, it would be of particular relevance to establish whether specific subgroups of acute myelogenous leukemia (AML) may benefit from ATRA-based treatments. In fact, AML is a very heterogeneous disease, which can be classified into subgroups according to the presence/absence of specific genetic alterations. The few available clinical trials on the use of ATRA in AML other than APL have provided conflicting results^3–5^.

Precision medicine requires the development of diagnostic tools capable of identifying individuals and groups of patients who may be sensitive to the therapeutic activity of specific agents. In a recent study, we developed a gene-expression model, consisting of 21 genes (*ATRA-21*), capable of correctly predicting ATRA sensitivity across a large panel of breast cancer cell lines characterized for their basal gene-expression profiles at the whole-genome level^6^. Despite the original development in the context of breast cancer^7^, application of *ATRA-21* to the over 400 cell lines derived from different types of leukemia and tumors present in the GDSC (Genomics of Drug Sensitivity in Cancer) database and profiled for their in vitro sensitivity to ATRA demonstrates that the predictive capability of the model is tumor-type independent^6^. In addition, the model correctly predicts ATRA sensitivity in the APL patients belonging to the TCGA (The Cancer Genome Atlas) and LEUCEGENE databases^6^.

A recent randomized clinical study (AMLSG 07-04) demonstrates that addition of ATRA to intensive treatment exerts a beneficial effect in ELN-favorable-risk and CEBPA-biallelic mutation patients^8^. In addition and contrary to the initial hypothesis of the trial, ATRA lacks therapeutic efficacy in NPM1-mutated cases. The present study reports the ATRA sensitivity predictions derived from our *ATRA-21* model in 1289 AML patients (Fig. 1a, Supplementary Methods, Table S1 and Figure S1). We apply the *ATRA-21* model to the adult AML cases contained in the TCGA, LEUCEGENE and GSE14468 data sets. In addition, we evaluate the pediatric AML cases of the TARGET database. The AML cases are classified according to the FAB (French American British) criteria, the ELN (European Leukemia Network)-RISK categories, and the presence/absence of clinically relevant genetic rearrangements as well as gene mutations. We compare the average *ATRA-21* predictions obtained in the various AML subgroups against the remainder of the entire AML population of the four data sets and estimate the effect size. Following clustering of the cases according to the FAB classification, the results obtained confirm that M3 (PML-RARα^+^) patients are endowed with significantly higher average *ATRA-21* values than the remainder of the AML population. On the basis of the *ATRA-21* values, AML cases belonging to the favorable ELN-RISK class are predicted to be particularly responsive to ATRA. As for the AML classification based on the presence/absence of the most prevalent genetic rearrangements, the cases showing an inversion of chromosome 16 [*inv(16)*] or a *t(8;21)* chromosomal translocation are characterized by *ATRA-21* predictions well above the average of the remainder AML population. With respect to this, it is interesting to notice that a case report study demonstrates that ATRA induces one partial and three complete remissions in four *t(8;21)* AML patients initially misdiagnosed as APL cases^9^. As for the AML subtypes defined by recurrent gene mutations, the only cluster predicted to be responsive to ATRA belongs to the larger group of patients characterized by biallelic mutations of the *CEBPA* gene. Indeed, AML cases presenting with *CEBPA-*biallelic mutations can be further divided into two relatively homogeneous subgroups on the basis of a gene expression profile (GEP) consisting of 95 genes (*CEBPA*^*MUT/GEP+*^ and *CEBPA*^*MUT/GEP−*^)^10^. The *CEBPA*^*MUT/GEP+*^ cases, which are endowed with this specific GEP, are predicted to be ATRA-sensitive on the basis of the *ATRA-21* score. In contrast, *CEBPA*^*MUT/GEP−*^ cases are characterized by very low *ATRA-21* predictions. As for the data obtained in AML patients with recurrent mutations, it is noticeable that NPM1-mutated AML do not show higher than average *ATRA-21* values. Taken together, the predicted responsiveness of *CEBPA*^*MUT/GEP+*^ AML patients to ATRA and the lack of predicted ATRA sensitivity in NPM1-mutated AML are in accordance with the findings of the AMLSG 07-04 study in which ATRA was added to intensive chemotherapy^8^. As a last remark, it should be emphasized that the favorable *ELN-risk* category encompasses the *t(8;21), inv(16),* mutated-*NPM1* without *FLT3-ITD* (normal karyotype) and mutated-CEBPA (normal karyotype) subgroups. Clear experimental validation of these results is required and the approach that we are currently pursuing involves exposure of primary blasts obtained from AML patients to ATRA in vitro. Nevertheless, it must be emphasized that the validity of the predictions on ATRA sensitivity based on our model is fully supported by the results obtained in the two above-mentioned clinical studies^8,9^. Finally, it should be mentioned that it is entirely possible that our analysis may overlook specific subgroups of patients or individual cases that may be sensitive to ATRA. In fact, as observed in solid tumors^6,7^, even AML types characterized by low-predicted average sensitivity to ATRA include a proportion of cases that may be sensitive to the retinoid.

**Fig. 1:**
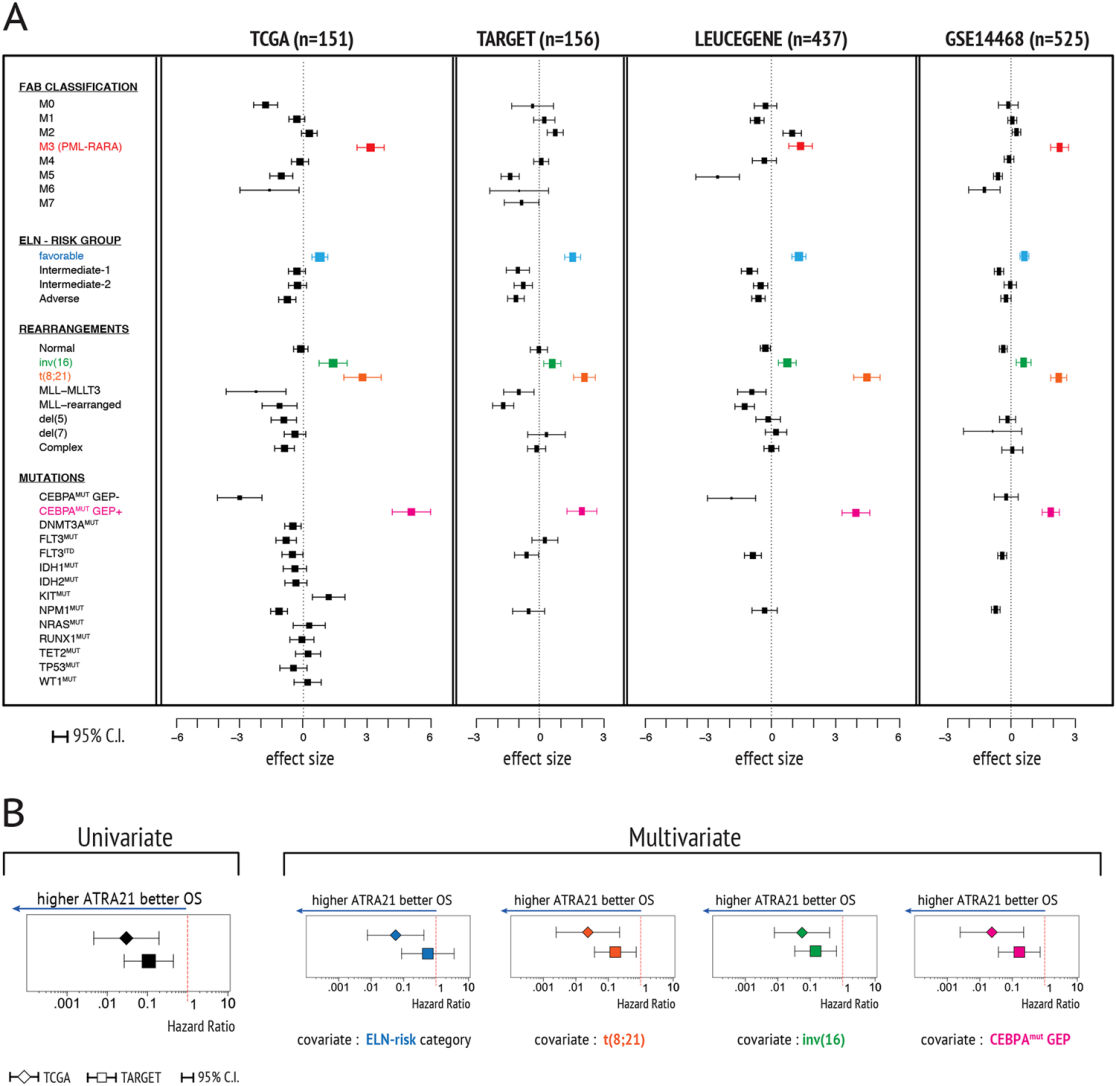
Predictions of ATRA sensitivity and prognostic potential of ATRA-21 in AML patients. **a** All AML samples were stratified according to the FAB classification (Top). Further stratifications according to the ELN-RISK groups, chromosomal rearrangements, and gene mutations were performed on non-APL AML patients (bottom). Forest plots of the standardized mean-difference effect size of each comparison are illustrated. The horizontal lines indicate the 95% interval of confidence. **b** Hazard ratio associated with one unit increase of ATRA-21 in AML patients of the TCGA (diamonds) and TARGET (squares) after univariate and multivariate Cox Proportional Hazard analysis of overall survival (OS)

Analysis of the overall survival data available in the TCGA and TARGET data sets is in line with the idea that *ATRA-21* may be endowed with prognostic potential (Fig. 1b). Indeed, univariate Cox Proportional Hazard analysis demonstrates a Hazard ratio significantly lower than one in both data sets. The same is true if multivariate analysis is performed using *ELN-RISK* categories, *CEBPA*^*MUT*^ status, and *t(8;21)* or *inv(16)* genetic aberrations as covariates. Statistical significance is observed for all the analyses with the exception of the *ELN-RISK* covariate in the TARGET data set. The observed associations between *ATRA-21* and overall survival suggest that the endogenous levels of ATRA may have an inhibitory role in the growth and progression of the subgroups of AML, which are predicted to be sensitive to the retinoid. We described similar associations in other tumor types, which are predicted to be responsive to ATRA on the basis of the *ATRA-21* model^6^.

To define the relative importance of all the single genetic aberrations and mutations observed in the AML population for the *ATRA-21* predictions, we trained a conditional decision model in the TCGA data set. The other three data sets were used to confirm the validity of the decision tree (Fig. 2). The purpose of this type of analysis is the identification of single markers, which may be used in the clinics for the selection of AML patients benefiting from ATRA-based treatments, when gene-expression data permitting the measurement of *ATRA-21* are not available. Correctly and expectedly, the PML-RARα translocation is identified as the most important factor that can be used to establish ATRA sensitivity. The factor ranking second for its importance is *CEBPA*^*MUT/GEP+*^. Indeed, *CEBPA*^*MUT*/*GEP+*^ and PML-RARα^+^ AML cases are characterized by average *ATRA-21* predictions, which are almost superimposable in all the data sets. The third and fourth factors are the *t(8;21)* and *inv(16)* genomic aberrations, respectively. However, it is noticeable that *inv(16)* patients show a large dispersion of the prediction values, which suggests that determination of *ATRA-21* in this subgroup may be useful to better discriminate retinoid responsive patients. The vast majority of the remainder groups of AML cases present with low-predicted sensitivity to ATRA. However, in these groups too, measurement of *ATRA-21* may be of use, as there are individual patients, possibly among the FLT3 wild-type cases, which show high values of this parameter.

**Fig. 2:**
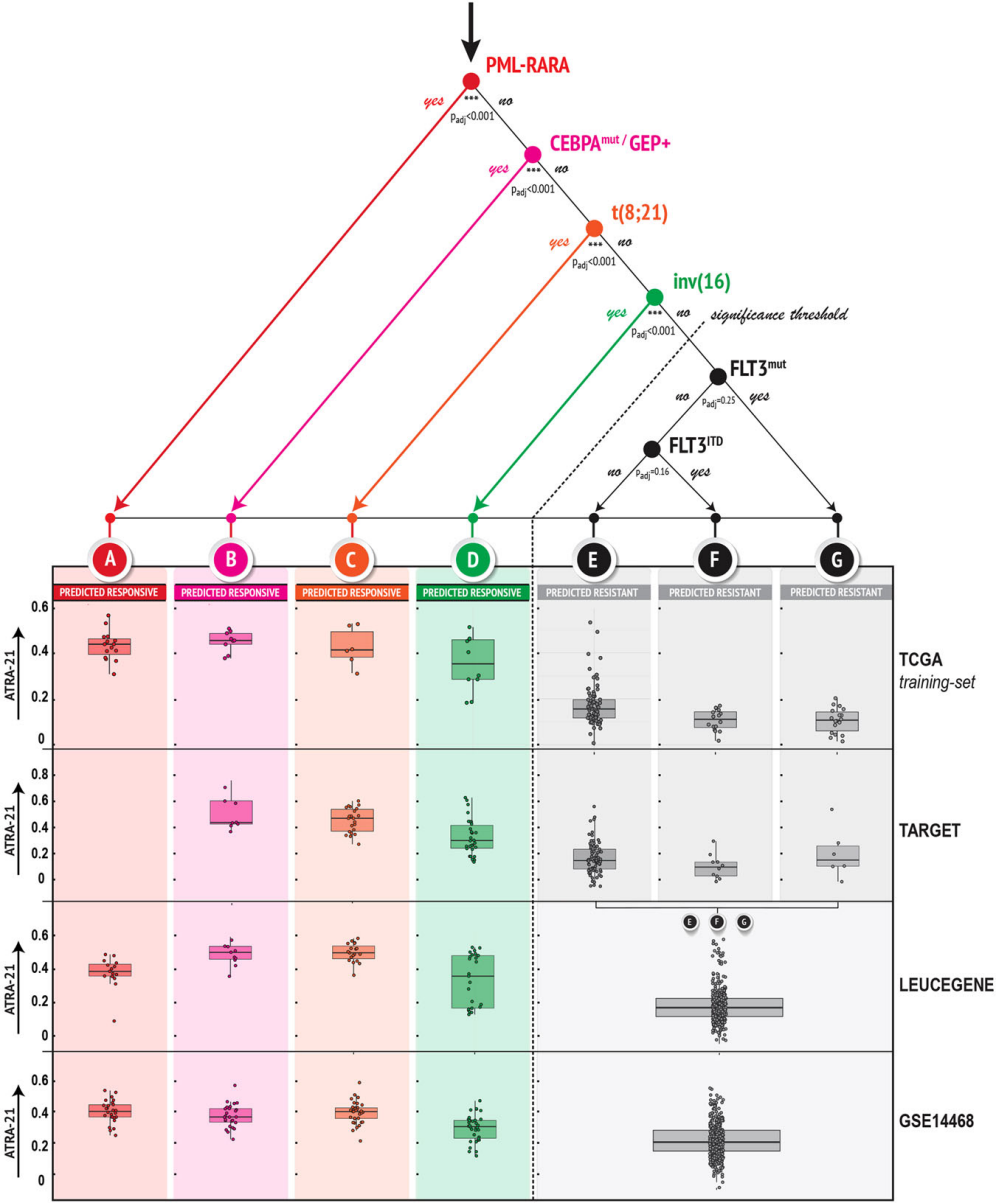
Decision tree for the selection of patients with predicted sensitivity to ATRA. A conditional inference-tree (top) was generated by linking AML cytogenetic and molecular characteristics to ATRA-21 predictions. The tree was built in the TCGA data set by taking into consideration: FAB-subtype; presence/absence of PML-RARA; BCR-ABL1; CBFB-MYH11; RUNX1-RUNX1T1; GATA2-MECOM; DEK_NUP214; MLLT3_KMT2A; other KMT2A rearrangements; mutations in KIT; TET2; WT1; TP53; NRAS; IDH1; IDH2; CEBPA(GEP^+/−^); NPM1; RUNX1; DNMT3A; FLT3 and presence of FLT-ITD. *P* values of the identified splits were adjusted using Bonferroni correction. Boxplots of the *ATRA-21* predictions in the seven identified subgroups (A–G) in the four data sets, are illustrated at the bottom

In conclusion, our predictions support the idea that other AML subtypes, besides the expected APL group, are likely to be responsive to the antiproliferative effects of ATRA. Indeed, we identify three new classes of AML with high *ATRA-21* predictions, i.e., biallelic *CEBPA* in its typical form or the GEP^+^ atypical form, *t(8;21)* and *inv(16)*. Each marker is sufficient to define predicted sensitivity to ATRA, irrespective of other concurrent genetic alterations. The validity of the *ATRA-21* model developed to predict ATRA sensitivity is confirmed by the results obtained in the clinics for AML patients. In addition, the data obtained support the idea that the *ATRA-21* model may have prognostic relevance in the context of AML, given the observed significant associations between *ATRA-21* values and overall survival data. In conclusion, the study represents the basis for further clinical trials aimed at evaluating the therapeutic advantage achievable with the addition of ATRA to standard therapy in the above groups of AML patients. In a precision medicine perspective, *ATRA-21* is likely to represent a new tool for the selection of AML patients who may benefit from treatments based on the use of ATRA.

## Supplementary information

Supplementary Information

Supplementary Table 1
