# Supplementary material for: The ATRA-21 gene-expression model predicts retinoid sensitivity in CEBPA double mutant, t(8;21) and inv(16) AML patients
Source: Blood Cancer J. 2019 Sep 30;9(10):76. doi: 10.1038/s41408-019-0241-5 (PMC6769013; doi:10.1038/s41408-019-0241-5)
Supplement: Supplementary file 1 — Supplementary Information [file 41408_2019_241_MOESM1_ESM.pdf]

Title: The ATRA-21 gene-expression model predicts retinoid sensitivity in CEBPA double mutant, t(8;21) and inv(16) AML patients

Marco Bolis, Mineko Terao, Linda Pattini, Enrico Garattini and Maddalena Fratelli

SUPPLEMENTARY INFORMATION

SUPPLEMENTARY METHODS

***ATRA-21* predictions in TCGA, TARGET, LEUCEGENE and GSE14468 datasets**

Gene-expression data (RNA-Seq) for the TCGA and TARGET datasets were retrieved from the *Genomics Data Commons* portal (GDC; https://gdc.cancer.gov) in the form of gene-counts (HT-Seq[1]) and further processed in the R statistical environment. Raw-counts were corrected for their TMM-adjusted library sizes (*edgeR*,[2]) and then logarithmically transformed using the *voom* [3] function included in the *limma* package. Gene-expression data (RNA-Seq) for the LEUCEGENE dataset were retrieved from NCBI-GEO (Part1: GSE49642; Part2: GSE52656; Part3: GSE62190; Part4: GSE66917; Part5: GSE67039). All samples were re-processed starting from raw sequencing reads as described in [4]. Replicated runs deriving from the same samples were merged. As for TCGA and TARGET datasets, gene counts (*hg38*) were corrected for their TMM-adjusted library sizes and then logarithmically transformed (*voom/limma*). Expression data were finally centered (*caret* package: caret.r-forge.r-project.org) and ATRA-sensitivity was predicted by applying the *ATRA-21* ridge regression model [4]. As only AML samples are considered in the study, each dataset was normalized separately, which differed from the pan-cancer approach described in Bolis et al. [4] where all tumor samples were normalized together.

Although *ATRA-21* was developed for gene-centric RNA-Seq data, it can be successfully applied to predict ATRA-sensitivity from microarray experiments [4], despite their lower dynamic range. Gene-expression data for *GSE14468* were retrieved from NCBI-GEO (Affymetrix Human Genome U133 Plus 2.0 Arrays). Raw microarray intensities were imported in the R statistical environment, background corrected and quantile normalized (*ReadAffy* and *rma* functions included in *affy* package) [5]. Probe intensities were summarized at the gene-level. Of the 21-genes needed to predict ATRA-sensitivity using the *ATRA-21* model, 3 are not included in the microarray platform (RP11-697H9, HOXA9 and HOXA10-AS). By taking advantage of the *ATRA-21* co-expression network identified in Bolis et al. [4], we replaced the 3 missing genes using their most significantly

co-expressed gene-neighbors (HOXA10 for both HOXA9 and HOXA10-AS, and ATL3 for RP11-697H9). As for the RNA-Seq datasets, centered expression data were ultimately used to predict ATRA-sensitivity by applying the *ATRA-21* ridge regression model.

### *Samples Annotation*

Available clinical and cytogenetic information for all patients included in the TCGA- and TARGET- cohorts were entirely retrieved from *cBioPortal* [6] and *Genomics Data Commons* (https://gdc.cancer.gov), respectively. Annotations for the samples included in the LEUCEGENE datasets were retrieved from various sources [7] [8] [9] [10]. The presence of the PML-RARA fusion gene was determined using the *FusionCatcher* (https://github.com/ndaniel/fusioncatcher) algorithm applied to raw *fastq* files. Annotations for the GSE14468-cohort were retrieved from Wouters et al. [11] and Taskesen et al. [12].

ELN-RISK groups were determined based on karyotype information, genetic rearrangements and mutations as defined in Mrozek et al. [13]. As detailed mutational information for *CEBPA* mutants (i.e. bi-allelic vs mono-allelic) was not available for all the datasets considered, we decided to apply the gene-expression signatures defined in Lavallée et al. [7] and Wouters et al. [11] for RNA-Seq and microarray samples, respectively. These signatures define a distinctive gene-expression profile ($GEP^{+}$), which is associated to JAK/STAT activation and uniform JAK inhibitor sensitivity [7, 14]. This characteristic profile is shared by all typical *CEBPA* bi-allelic mutants and by certain atypical *CEBPA* bi-allelic mutants. The remaining atypical *CEBPA* bi-allelic mutants are not endowed with this characteristic gene-expression profile ($GEP^{-}$). These samples have a gene-expression pattern similar to *CEBPA* mono-allelic mutants and do not show uniform sensitivity to JAK inhibitors. In this work, we define two classes of *CEBPA* mutants: $CEBPA^{mut/GEP+}$ and $CEBPA^{mut/GEP-}$. The first group includes all typical CEBPA bi-allelic mutants and $GEP^{+}$ atypical bi-allelic mutants, while the second group includes mono-allelic mutants and $GEP^{-}$ atypical bi-allelic mutants.

***Forest plots***

All samples included in the 4 AML datasets were assigned to subgroups according to the FAB-classification, ELN-RISK group, cytogenetic rearrangements and genetic mutations. In all datasets separately, we compared the *ATRA-21* predictions of each sub-group to those of all the other samples. The standardized mean-difference effect size of each comparison was computed in the R statistical environment using the *esc* library (https://cran.r-project.org/package=esc). Forest plots with 95% confidence intervals were generated using the *metafor* package (http://www.metafor-project.org).

***Conditional Inference Tree***

A conditional inference-tree was generated in the R statistical environment (*ctree,* party package: *http://party.R-forge.R-project.org*) by linking sample-characteristics and *ATRA-21* predictions. The tree was built in the TCGA dataset by taking into consideration: FAB-subtype; presence/absence of PML-RARA; BCR-ABL1; CBFB-MYH11; RUNX1-RUNX1T1; GATA2-MECOM; DEK_NUP214; MLLT3_KMT2A; other KMT2A rearrangements; mutations in KIT; TET2; WT1; TP53; NRAS; IDH1; IDH2; CEBPA(GEP$^{+/-}$); NPM1; RUNX1; DNMT3A; FLT3 and presence of FLT-ITD. Significance of the identified splits was adjusted by using Bonferroni correction. The decision-tree was built using the most comprehensive dataset (TCGA), and then tested on TARGET, LEUCEGENE and GSE14468 samples.

***Univariate and multivariate survival analysis***

The impact of ATRA-21 on overall survival in the TCGA and TARGET datasets (the only datasets that included overall survival information) was determined by the Cox Proportional Hazard regression using the survival package in R. For multivariate analysis we considered separately

ELN-RISK categories, RUNX1-RUNX1T1, CBFB-MYH11 and CEBPA mutation status as covariates.

SUPPLEMENTARY REFERENCES


1. Anders S, Pyl PT, Huber W: HTSeq--a Python framework to work with high-throughput sequencing data. Bioinformatics 2015, 31(2):166-169.

2. Robinson MD, McCarthy DJ, Smyth GK: edgeR: a Bioconductor package for differential expression analysis of digital gene expression data. Bioinformatics 2010, 26(1):139-140.

3. Law CW, Chen Y, Shi W, Smyth GK: voom: Precision weights unlock linear model analysis tools for RNA-seq read counts. Genome Biol 2014, 15(2):R29.

4. Bolis M, Garattini E, Paroni G, Zanetti A, Kurosaki M, Castrignano T, Garattini SK, Biancardi F, Barzago MM, Gianni M et al: Network-guided modelling allows tumor-type independent prediction of sensitivity to all-trans retinoic acid. Ann Oncol 2016.

5. Gautier L, Cope L, Bolstad BM, Irizarry RA: affy--analysis of Affymetrix GeneChip data at the probe level. Bioinformatics 2004, 20(3):307-315.

6. Cerami E, Gao J, Dogrusoz U, Gross BE, Sumer SO, Aksoy BA, Jacobsen A, Byrne CJ, Heuer ML, Larsson E et al: The cBio cancer genomics portal: an open platform for exploring multidimensional cancer genomics data. Cancer Discov 2012, 2(5):401-404.

7. Lavallee VP, Krosl J, Lemieux S, Boucher G, Gendron P, Pabst C, Boivin I, Marinier A, Guidos CJ, Meloche S et al: Chemo-genomic interrogation of CEBPA mutated AML reveals recurrent CSF3R mutations and subgroup sensitivity to JAK inhibitors. Blood 2016, 127(24):3054-3061.

8. Lavallee VP, Lemieux S, Boucher G, Gendron P, Boivin I, Armstrong RN, Sauvageau G, Hebert J: RNA-sequencing analysis of core binding factor AML identifies recurrent ZBTB7A mutations and defines RUNX1-CBFA2T3 fusion signature. Blood 2016, 127(20):2498-2501.

9. Celton M, Forest A, Gosse G, Lemieux S, Hebert J, Sauvageau G, Wilhelm BT: Epigenetic regulation of GATA2 and its impact on normal karyotype acute myeloid leukemia. Leukemia 2014, 28(8):1617-1626.

10. Analyse préliminaire du rôle des "Ubiquitin specific peptidases" et de l'axe USP7-MDM2-TP53-CDKN1A dans les leucémies myéloïdes aiguës

11. Wouters BJ, Lowenberg B, Erpelinck-Verschueren CA, van Putten WL, Valk PJ, Delwel R: Double CEBPA mutations, but not single CEBPA mutations, define a subgroup of acute myeloid leukemia with a distinctive gene expression profile that is uniquely associated with a favorable outcome. Blood 2009, 113(13):3088-3091.

12. Taskesen E, Bullinger L, Corbacioglu A, Sanders MA, Erpelinck CA, Wouters BJ, van der Poel-van de Luytgaarde SC, Damm F, Krauter J, Ganser A et al: Prognostic impact, concurrent genetic mutations, and gene expression features of AML with CEBPA mutations in a cohort of 1182 cytogenetically normal AML patients: further evidence for CEBPA double mutant AML as a distinctive disease entity. Blood 2011, 117(8):2469-2475.

13. Mrozek K, Marcucci G, Nicolet D, Maharry KS, Becker H, Whitman SP, Metzeler KH, Schwind S, Wu YZ, Kohlschmidt J et al: Prognostic significance of the European LeukemiaNet standardized system for reporting cytogenetic and molecular alterations in adults with acute myeloid leukemia. J Clin Oncol 2012, 30(36):4515-4523.

14. Tyner JW: JAKed up phenotype of CEBPA-mutant AML. Blood 2016, 127(24):2946-2947.

***Figure S1***

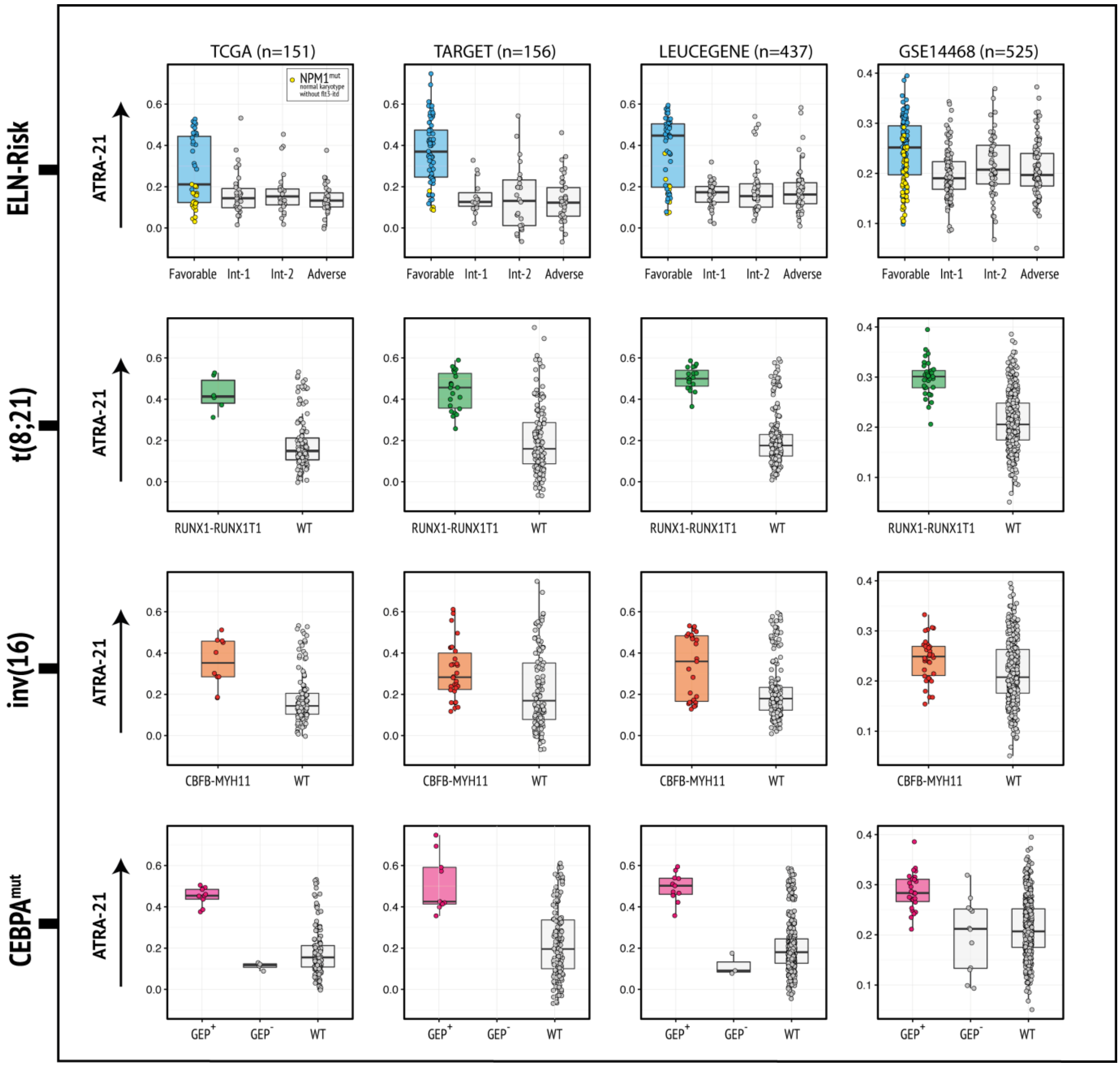


**Figure S1: *ATRA-21* predictions in the identified ATRA-sensitive subgroups**

Boxplots and individual *ATRA-21* predictions in the 4 AML datasets after classification for ELN-Risk, t(8;21), inv(16) and CEBPA mutational status. For favorable ELN-Risk category, NPM1-mutants are indicated in yellow.
